# Increased Adiposity as a Potential Risk Factor for Lower Academic Performance: A Cross-Sectional Study in Chilean Adolescents from Low-to-Middle Socioeconomic Background

**DOI:** 10.3390/nu10091133

**Published:** 2018-08-21

**Authors:** Paulina Correa-Burrows, Yanina Rodriguez, Estela Blanco, Sheila Gahagan, Raquel Burrows

**Affiliations:** 1 Institute of Nutrition and Food Technology, University of Chile, Santiago 7830490, Chile; yanirod77@hotmail.com (Y.R.); rburrows@inta.uchile.cl (R.B.); 2 Division of Child Development and Community Health, University of California San Diego, La Jolla, CA 92093, USA; esblanco@ucsd.edu (E.B.); sgahagan@ucsd.edu (S.G.)

**Keywords:** adiposity markers, obesity, fat mass, abdominal obesity, adolescent health, school performance

## Abstract

We explored the association between excess body fat and academic performance in high school students from Santiago, Chile. In 632 16-year-olds (51% males) from low-to-middle socioeconomic status (SES), height, weight, and waist circumference were measured. Body-mass index (BMI) and BMI for age and sex were calculated. Weight status was evaluated with 2007 World Health Organization (WHO) references. Abdominal obesity was diagnosed with International Diabetes Federation (IDF) references. Total fat mass (TFM) was measured with dual-energy X-ray absorptiometry (DXA). TFM values ≥25% in males and ≥35% in females were considered high adiposity. School grades were obtained from administrative records. Analysis of covariance examined the association of fatness measures with academic performance, accounting for the effect of diet and physical activity, and controlling SES background and educational confounders. We found that: (1) having obesity, abdominal obesity, or high adiposity was associated with lower school performance alone or in combination with unhealthy dietary habits or reduced time allocation for exercise; (2) high adiposity and abdominal obesity were more clearly related with lower school grades compared to obesity; (3) the association of increased fatness with lower school grades was more salient in males compared to females.

## 1. Introduction

Excess weight at a young age is associated with cardiometabolic disorders [1,2], reduced quality of life [3], and the risk of obesity and its comorbidities in adulthood [4]. Biological, behavioural, and environmental factors lead to excess weight gain and contribute to the rising prevalence of obesity worldwide. National surveys measuring height and weight in individuals across Chile indicate that at least one in three Chilean adolescents are either overweight or obese [5,6]. Of the OECD (Organisation for Economic Cooperation and Development) countries, Chile ranks sixth in overweightness and obesity among children aged 11–15 years [7]. 

National reports also indicate that excess weight and risk factors are more prevalent among socially vulnerable groups [5,6,8,9]. According to these surveys, low-to-middle SES groups have greater access to energy-dense diets and efforts to provide safe and convenient places for physical activity, particularly for low-income populations, are still insufficient [9]. As a result, adolescents from low-to-middle SES are at increased risk of being obese than their high-SES counterparts. Several studies show that SES has its most crucial impact on health during adolescence [10,11]. The so-called ‘adolescent-emergent model’ suggests that the SES–health relationship is weak in early childhood but then strengthens with age, due to determinants such as peer influence, personality, and autonomous decision-making [10,11,12]. For instance, physical activity is more strongly correlated with SES during adolescence than earlier in childhood [11]. This model states that children begin to model the health behaviours of their parents early in life, but as they grow older, the extent to which these behaviours are internalized and pursued by the youth will be more influential to their health. 

The cardiometabolic complications of childhood and adolescent obesity have been widely explored; however, its impact on cognition and educational outcomes has recently received a lot of scientific attention. Certain brain structures continue to develop throughout adolescence; therefore, whether obesity impacts the adolescent brain is highly relevant. Frontal and temporal lobe abnormalities as well as reduced hippocampal volume were found in obese adolescents [13]. Both the prefrontal cortex (PFC) and the hippocampus play pivotal roles in the cognitive abilities (i.e., learning, memory, and cognitive control) that are needed to perform well in school [14]. Also, obesity in youths is associated with poorer executive functioning skills, such as inhibitory control and working memory, which are critical for academic achievement [15]. Furthermore, obesity-associated biomarkers, including gut hormones (i.e., ghrelin, GLP-1), adipokines (i.e., leptin), and proinflammatory cytokines (i.e., TNF-α, CRP), have been linked with cognition and memory [15]. Abnormally high leptin levels, which are largely related to adiposity, limit the potential for synaptic plasticity and trafficking of neurotransmitter receptors in the hippocampus and might be responsible for some memory deficits [16]. In adolescents, hyperleptinemia was associated with lower academic performance in high school [17] and on tests for college admission [18].

Because paediatric obesity has been related to specific outcomes that are relevant for children’s educational attainment, some authors have examined the association of weight status with academic attainment as measured by school grades and standardised test scores. A negative, significant association was reported for adolescent students in Finland [19], Iceland [20], Iran [21], Portugal [22], South Korea [23], the U.S. [24,25], and the United Kingdom [26]. 

Most studies addressing the academic implications of adolescent obesity rely on body-mass index (BMI) as the sole measure. With some exceptions [23,26,27], very few studies have used other measures of adiposity, despite evidence that fat tissue is metabolically active and produces proteins and inflammatory markers associated with learning, memory, and general cognitive function [15,28]. Furthermore, systematic reviews suggest that existing studies are not representative of developing countries [29,30], where overweight and obesity are steadily increasing in children and youths. A focus on academics might have important preventive implications as academic outcomes are linked to expectations of better college opportunities and future job status and income. In this study, we explored the association between excess body fat and academic performance in Chilean high school students. This paper aimed to explore the association between excess body fat and academic performance in high school students from Santiago, Chile. Because Chilean adolescents of low-to-middle SES are at greater risk of excessive weight gain, and because the SES effects on health status would be stronger in years where the child is making conscious decisions about physical activity and healthy eating, we concentrated on adolescents from low-to-middle socioeconomic backgrounds. We hypothesised that adolescents with obesity, abdominal obesity, or high adiposity would have lower school grades and lower grade-point average (GPA). 

## 2. Materials and Methods

### 2.1. Study Design and Participants

This was a cross-sectional study within an infancy cohort. The sample was made up of 16–17-year-old adolescents living in Santiago, Chile, from low-to-middle SES. Participants were recruited at 4 months from public healthcare facilities in the southeast area of Santiago (*n* = 1791). They were born at term of uncomplicated vaginal births, weighed >3.0 kg, and were free of acute or chronic health problems. At 6 months, infants free of iron deficiency anemia (*n* = 1657) were randomly assigned to receive iron supplementation or no added iron (ages 6–12 months). They were assessed for developmental outcomes in infancy and at 5, 10, and 15 years [31]. At 16–17 years, those with complete data in each wave (*n* = 679) were also assessed for obesity risk and the presence of cardiovascular risk factors in a half-day evaluation that included assessment of dietary habits and nutritional content of food intake. Of them, *n* = 632 (93%) entered high school and were eligible for this study. Ethical approval was obtained by the institutional review boards of the University of Michigan, Institute of Nutrition and Food Technology (INTA), University of Chile, and the University of California, San Diego. Participants and their primary caregiver provided informed and written consent, according to the norms for Human Experimentation, Code of Ethics of the World Medical Association (Declaration of Helsinki, 1995).

### 2.2. Measurements

#### 2.2.1. School Grades

School grades in high school (9th to 12th) and final grade-point average (GPA) were collected from administrative records of the Ministry of Education (Chile). Since schools may have differed in grading policies, grades (on a scale of 1–7) were transformed into scores (range of 210–825), following the Ministry of Education criteria. The arithmetic average of each subject taken during each academic year was calculated and the result was compared in the conversion table provided by the Department of Assessment, Measurement and Educational Record, University of Chile, which complies with specifications on behalf of the Ministry of Education. The same procedure was used to convert the GPA into a score. Both school grades and GPA were used as continuous variables. 

#### 2.2.2. Anthropometric Assessment and Definitions

A physician used standardized procedures to measure the adolescent’s height (cm) to the nearest 0.1 cm, using a Holtain stadiometer, and weight (kg) to the nearest 0.1 kg, using a scale (Seca 703, Seca GmbH & co. Hamburg, Germany). Waist circumference (WC) was measured with nonelastic flexible tape and recorded to 0.1 cm (Seca 201, Seca GmbH & co. Hamburg, Germany). Measurements were taken twice, with a third measurement if the difference between the first two exceeded 0.3 kg for weight, 0.5 cm for height, and 1.0 cm for WC. BMI and BMI for age and sex (BMI*z*) were calculated. Weight status was evaluated according to WHO references: normal weight (BMI*z* from −1 to 1 SD); excess weight (BMI*z* > 1 SD). Abdominal obesity was defined as WC ≥ 90 cm for males and ≥80 cm for females, according to the International Diabetes Federation (IDF). Total fat mass (TFM) was determined via dual-energy X-ray absorptiometry (DXA) (apparatus: Lunar Prodigy Corp. Software: Lunar iDXA ENCORE 2011, Version 13.60.033, Madison, WI, USA.). Because there is no generally accepted definition of obesity based on TFM, we used cutoffs by Taylor et al.: values ≥25% in males and ≥35% in females were considered high adiposity [32].

#### 2.2.3. Dietary Assessment at Age 16 Years

Nutritional quality of diet was measured considering the amount of saturated fats and sugars in the foods consumed during breakfast, lunch, dinner, snacks at school, and snacks at home within the past three months. Assessment was performed with a food frequency questionnaire (FFQ), validated using three 24-h recalls to include weekends (Spearman’s rank correlation coefficient between the FFQ and the 24-hour dietary recalls exceed 0.35 in two-thirds of the food items, and all correlation coefficients were significant) [33,34]. A list of 120 foods and beverages was used. The frequency of food consumption was assessed by a multiple response grid; respondents were asked to estimate how often a particular food or beverage was consumed. Categories ranged from ‘never’ to ‘five or more times a week’. The electronic version of the Chilean Food Composition Tables/Database was used to assess the quality of the foods’ composition [35]. Food items were classified as high in saturated fats and sugars, high in sugars although low in saturated fats, and nutrient-rich foods. We assigned adjustment weights to each food item conditioned to its nutritional quality. A continuous score ranging from 0–10 was computed by adjusting the frequency of food consumption to the nutritional quality, with higher scores representing healthier dietary habits. We applied quartile cutoffs for the Chilean adolescent population (comprising students of high-, middle-, and low-SES) to classify the nutritional quality of the overall diet of participants into three groups: unhealthy (≤4.3 or ≤25th percentile), fair (from 4.4 to 5.9 or >25th percentile and <75th percentile), and healthy (≥6.0 or ≥75th percentile) [33].

#### 2.2.4. Physical Activity at Age 16 Years

We approached physical activity (PA) with scheduled, repetitive, and planned PA, accounting for the number of weekly hours devoted to physical education (PE) and extracurricular sports. To measure this, we used a questionnaire that was validated in a previous study using accelerometry-based activity monitors in both elementary and high school children [36]. The questionnaire was administered by a researcher to all students at the time they attended the anthropometric examination. Participants were asked: (1) On average, over the past week, how often did you engage in PE and extracurricular sports? (2) On those days, on average, how long did you engage in such activities? With this information, we estimated the average hours per week of scheduled PA. Participants having ≤90 minutes of weekly scheduled PA, which is the mandatory time for school-based PE in Chile, were considered to be physically inactive.

#### 2.2.5. Socioeconomic Background

The present study used family structure and parental education level as proxies of SES. Parental education is an important predictor of children’s educational outcomes [37]. In infancy, participants’ parents reported the highest schooling level they have been enrolled in, and also the highest grade they completed at that level. Five hierarchic levels were defined: (1) no education completed, (2) first level (primary school), (3) secondary level (first phase or 9th–10th), (4) secondary level (second phase or 11th–12th), and (5) post-secondary nontertiary educations [38]. Then, we merged these categories into two: incomplete secondary education (1 + 2 + 3), and complete secondary education or higher (4 + 5). Also, because a relationship between children’s educational outcomes and family structure has been described [39], we included a variable denoting whether the participant was raised in a fatherless family. This information was reported by the participant’s mother or legal guardian. Last, to control for potential design biases, we used a categorical variable denoting whether the participant had received iron supplementation or no added iron at 6–12 months.

#### 2.2.6. Type of Secondary Education

In Chile, secondary education includes academic high schools, which provide theoretical education in languages, mathematics, history, and sciences; vocational training schools; and adult schools. Data on the type of secondary education attended by participants was retrieved from publicly available records at the Ministry of Education. 

#### 2.2.7. Iron Supplementation in Infancy

Aiming to control for potential design biases, we used a categorical variable denoting whether the participant had received iron supplementation or no added iron at 6–12 months.

### 2.3. Statistical Analysis

Student’s *t-*test and chi-squared test were used for comparison of continuous and categorical variables, respectively, in male and female participants. Due to sex differences in fatness and academic attainment, we ran separate analyses for male and female participants. To examine the association of excess weight, abdominal obesity, and high adiposity (exposure) with school performance (outcome), we conducted analysis of covariance (ANCOVA). Each fatness measure was tested against school grades (9th to 12th) and overall GPA using two models. Model 1 was unadjusted. Model 2 added parental education, family structure, age at high school completion, type of secondary education (vocational and adult school), and a variable denoting iron supplementation in infancy (no added iron at 6–12 months). Because diet and physical activity have been found to be associated with academic achievement in studies conducted in Chile [40,41,42], all models included interactions of fatness measures with diet as well as interactions of fatness with time allocation for PA. Last, the effect size (ES) for difference was estimated using Cohen’s *d* coefficients. Data were analyzed using Stata for Windows version 15.0 (Lakeway Drive College Station, TX, US). A *p* value of 0.05 was used to test for statistical significance.

## 3. Results

### 3.1. Description of the Sample

Of the adolescents (*n* = 679) who participated in a prior obesity/cardiovascular study, 632 (93%) entered high school and were included in the present study. A comparison of the participants included in and excluded from this study is shown in the Supplementary Materials (Table S1). The final sample was comprised of adolescents (51% males) with an average age of 16.8 years (SD: 0.3). These adolescents completed high school at an average age of 18.5 years (SD: 0.8). Thirty-eight percent had excess weight, 32.6% had abdominal obesity, and 44.3% had high adiposity (Table 1). Forty percent of adolescents came from a fatherless home, and one-third had a mother with incomplete secondary education. As for participants’ lifestyles, 21% were regarded as having an unhealthy diet, while 58% were considered to be physically inactive. Last, 51% of our participants attended a vocational high school. 

After controlling for sex, we found that female adolescents had better school performance than males (*p* < 0.001). While the prevalence of obesity was similar in both groups, the prevalence of abdominal obesity (*p* < 0.001) and high adiposity (*p* < 0.001) was significantly higher in females. As for SES background, we found that females were more likely to have a father with incomplete higher education (*p* < 0.001). They were also more likely to be physically inactive compared to males (*p* < 0.001), although no differences were observed in relation to diet quality.

### 3.2. Relationship of Increased Adiposity with Academic Attainment, Accounting for the Effect of Diet Quality

When we explored the association of fatness with school grades, accounting for the effect of diet quality, we observed that in both males and females, the greatest losses in school attainment were related to the combined effect of increased fatness and poor diet quality. In a first analysis, where fatness was approached with obesity (Table 2), the combined effect of obesity and unhealthy dietary habits was associated with lower school grades across high school (grades 9th to 12th and overall GPA) in both sexes. The ES for the difference in males indicates that the combined effect of obesity and poor diet quality was moderate to large (Cohen’s *d* ranging from 0.51 to 0.67) in males, whereas in females, the effect was moderate (Cohen’s *d* ranging from 0.33 to 0.45). In males who had a fair-to-healthy diet, but still were obese, obesity was related to lower school attainment across high school, whereas in females who had a fair-to-healthy diet, but still were obese, obesity was associated with lower school performance only in grades 11 and 12. As for the effect of diet quality in nonobese adolescents, we observed that in males, a poor diet quality was related to lower school attainment in grades 11 and 12. A lower GPA was also observed in nonobese males who had unhealthy diets. In nonobese females, unhealthy dietary habits were associated with lower school performance only in grade 11.

When the exposure was abdominal obesity instead of obesity, we found that the combined effect of fatness and unhealthy dietary habits on school grades was large in males (*d* values ranging from 0.69 to 0.74) and moderate-to-large females (*d* values ranging from 0.51 to 0.69) (Table 3). In participants having fair-to-healthy dietary habits, but still having abdominal obesity, fatness was associated with lower school grades and lower GPA compared to peers without abdominal obesity and fair-to-heathy diets. In both males and females, abdominal obesity had a moderate effect on school grades, as *d* coefficients ranged from 0.33 to 0.42 and 0.33 to 0.40 in males and females, respectively. In this model, unhealthy dietary habits in participants without abdominal obesity was related to lower school attainment in males and females, but the association was significant only in grades 10 and 12 for males, grade 11 for females, and GPA for both groups. The ES for the difference indicates a small effect of diet alone on school grades. 

In a third model, where fatness was measured with total fat mass percentage, we found that the combined effect of high adiposity and unhealthy dietary habits was associated with lower school performance in both sexes (Table 4). Cohen’s *d* coefficients indicate that the combined impact of diet and fatness on school grades was moderate in females and moderate to large in males. In males with high adiposity, although having fair-to-healthy dietary habits, we also observed lower school grades compared to males without high adiposity, although having fair-to-healthy diets. The ES for difference ranged from 0.36 to 0.39, denoting a moderate effect of high adiposity on school grades in males who had fair-to-healthy diets. In females with high adiposity, on the other hand, we only found a significant association of fatness with school attainment in grade 12 and GPA and a trend towards a lower school attainment in grade 11. As for the effect of unhealthy dietary habits in participants with normal adiposity, again, the association was more salient in males than in females. Males with normal adiposity and unhealthy diets significantly underperformed compared to males with normal adiposity and fair-to-healthy diets in the 10th, 11th, and 12th grades and in terms of GPA, whereas females with normal adiposity and unhealthy diets significantly underperformed compared to peers with normal adiposity and fair-to-healthy diets only in the 12th grade and in terms of GPA. 

### 3.3. Relationship of High Adiposity with Academic Attainment, Accounting for the Effect of Physical Activity

When we explored the association of fatness with school grades, accounting for the effect of PA on the academic outcome, we observed that in both males and females, the greatest losses in school attainment were related to the combined effect of increased fatness and physical inactivity. When fatness was approached with obesity (Table 5), the combined effect of obesity and reduced time allocation for exercise was associated with lower school grades across high school (grades 9 to 12 and overall GPA) in both sexes. The ES for difference in males indicates that the combined effect of obesity and physical inactivity was large, whereas in females, the effect was moderate. In males who devoted >90 min/week to exercise, obesity was related to lower school attainment across high school. In females who devoted >90 min/week to exercise, on the other hand, obesity was associated with lower school performance only in grade 12 and overall GPA. As for the effect of exercise in nonobese adolescents, we observed that in both sexes, a reduced time allocation for exercise was related to lower school performance, with the exception of grade 9. Nonobese males and females who were physically inactive significantly underperformed compared to nonobese peers who were physically active. In both groups, however, the ES for difference indicates that the effect of reduced time allocation for exercise on school grades was small (*d* < 0.30).

When the exposure was abdominal obesity instead of obesity, we found that the combined effect of fatness and physical inactivity on school grades was moderate in females and moderate to large in males (Table 6). In males and females with time allocation for exercise >90 min/week, obesity was related to lower school grades and the ES for difference indicates that the effect of abdominal obesity on school attainment was moderate in both sexes. In adolescents without abdominal obesity, the effect of physical inactivity on school grades was found to be significant only in the 12th grade (males) and in terms of GPA (males and females). 

Finally, when fatness was approached with total fat mass percentage, we found that the combined effect of high adiposity and physical inactivity on school grades was large in males and females (Table 7). In both sexes, for adolescents with time allocation for exercise >90 min/week, obesity was related to lower school grades; the ES for difference indicates that the effect of high adiposity on school attainment was small to moderate in females (*d* values ranging from 0.25 to 0.35) and moderate in males (*d* values ranging from 0.42 to 0.49). In adolescents with normal adiposity, the effect of physical inactivity on school grades was found to be significant only in the 12th grade (males) and in terms of GPA (males and females). 

## 4. Discussion

### 4.1. Main Findings

We examined the association of fatness measures with school performance in adolescents of low-to-middle SES in a nonindustrialised country. We found that having obesity, abdominal obesity, or high adiposity was associated with lower school performance alone or in combination with unhealthy dietary habits or reduced time allocation for exercise. Second, the findings show that high adiposity and abdominal obesity are more clearly related with lower school grades compared to obesity. Last, the results also indicate that the association of increased fatness with lower school performance was more salient in males than in females.

A number of studies have tested the association of academic outcomes with obesity and their results are similar to ours. In a sample of 6346 adolescents from Iceland, Kristjánsson et al. found that BMI, physical activity, and dietary habits were all independently associated with academic achievement [20]. In this study, self-esteem, which correlated positively with school grades, was negatively influenced by increasing levels of BMI. In Portuguese adolescents ranging in age from 12 to 14 years, cardiorespiratory fitness and weight status were independently and combined related to academic achievement in in mathematics, language (Portuguese), foreign language (English), and sciences [22]. In a sample of high school and college students from five regions across the U.S., McCann and Roberts found that even after controlling for demographic variables, intelligence, personality, and well-being, obese students obtained significantly lower grades than normal-weight students in the eighth grade, community college, and university [43]. Among South Korean adolescents in grades 7–12, overweightness/obesity was negatively associated with academic performance in both boys and girls. In both sexes, the odds of having poor and very poor school performance were substantially and significantly higher in overweight and obese students compared to normal-weight students. These association remain significant after accounting for the effect of sociodemographic determinants as well as the impact of frequency of muscle-strengthening exercises and vigorous and moderate PA [44]. Also, it is worth noting that other studies also show that overweightness/obesity affects a range of behaviours that may affect students’ performance, such as classroom behaviour [24], attendance, rate of drop-out [45,46], and academic adjustment [24]. Self-esteem, school satisfaction, and school connectedness have also been postulated as determinants of school performance and have been related with weight status in young populations [47]. 

A second major finding of our study has to do with the fact that abdominal obesity was the exposure showing the more clear association with school performance, followed by high adiposity and obesity. A *d* value from 0.3 to 0.6 corresponds with a moderate ES, whereas a *d* value greater than 0.6 corresponds with a large ES. Therefore, the effect of obesity on school performance tended to be moderate, yet the effect of abdominal obesity and high adiposity ranged from moderate to large. This suggests that BMI might be underestimating the true effects of obesity on the adolescent brain. Although BMI is the most commonly used method for diagnosing obesity, it has been criticized because it does not always reflect true body fatness, which may be better assessed using body fat percentage and distribution. This also seems to be the case when it comes to understanding how obesity impacts the adolescent brain, specifically the hippocampus and the prefrontal cortex. Adolescence is a period of rapid growth and maturation and is a particularly critical period for hippocampal development and for neural organisation and functional connectivity between the PFC and other brain areas. The hippocampus plays a role in the processing of short- to long-term memory, learning, and emotions, whereas the PFC is involved in executive functions, which allow conscious control, planning, time management, and organisation [11]. A reduced hippocampal and PFC size has been associated with obesity and obesity-related cardiometabolic complications, including insulin resistance and nonalcoholic steatohepatitis [8,13,48]. Excess adiposity also promotes chronic low-grade inflammation and elevated levels of proinflammatory cytokines in the brain, which have been linked with loss of hippocampal and PFC tissue [49]. Likewise, in these brain regions, obesity-induced oxidative stress prompts cell senescence, cytotoxicity, and apoptosis and inhibits the survival and growth of neurons [50]. Furthermore, excess adiposity impairs long-term potentiation (LTP), a process that enhances neural transmission and improves information processing, storage, and retrieval, and thereby promotes memory consolidation and learning [51]. Lastly, excess fat accumulation decreases the expression of hippocampal brain-derived neurotrophic factor [52], which also hampers the conversion of short-term potentiation into LTP. 

The fact that excessive fatness appears to take a special toll on males in terms of school performance is also of interest. Cross-sectional and longitudinal studies conducted in adolescents report results similar to ours. In a sample of Hispanic adolescents in grades 9–11 in Texas (U.S.), obese males had lower scores in both reading and mathematics as measured by the Texas Assessment of Knowledge and Skills (TAKS) in comparison to their nonobese counterparts. However, no relationship was found between obesity and TAKS performance in females [53]. Using data from the Longitudinal Study of Australian Children, which were linked to national test scores in numeracy and literacy, obesity and BMI were negatively related to cognitive achievement in boys (aged 8–12 years), but not in girls [54]. The effect could not be explained by family income, parental education, genetic background, or past cognitive achievement and was robust to different measures of adiposity. Additionally, in preadolescents from Brazil, a negative association between skinfold thickness and performance in mathematics was reported to be significant in males, but not in females [55]. Our findings might be consistent with those of the literature showing that men bear a heavier burden following excessive weight gain. Males with increased body fat have higher risk of metabolic syndrome and insulin resistance and have higher liver fat levels than females with the same condition [56]. In a previous study conducted on the same sample, the risk of cardiovascular complications was higher in obese males compared to obese females [2]. The risk was even higher in obese males with reduced muscle mass or augmented body fat accumulation [57]. This sexual dimorphism of obesity is likely related to the differential distribution of adipose tissue in males and females and hence entails important metabolic consequences. Males tend to accrue visceral fat, which has been correlated with increased cardiovascular risk. Premenopausal females, on the other hand, accumulate more fat in subcutaneous deposits, which may protect females from some of the negative consequences of obesity. Visceral fat increases the production of proinflammatory cytokines and interferes with the hormones that regulate appetite, mood, and cognitive function. 

### 4.2. Implications of These Results

Our findings have implications for public health and education policy and provide schools and parents with potentially significant incentives for encouraging the healthy weight of adolescents. Because academic results are a high priority for schools, educators, and parents, the relationship between students’ health and school success is important to disseminate. Healthy dietary habits, physical activity, and healthy weight in adolescents are important contributors towards good school performance [58]. Thus, the promotion of healthy lifestyles and good academic outcomes might share a common ground. A second implication of these results relates to the detrimental effects of excess weight gain during critical periods of brain development. Adolescents around the world are highly exposed to risk factors for overweightness and obesity, and thus may also be at risk of cognitive deficits. The adolescent period is particularly critical for hippocampal and PFC development, so the adolescent brain might be particularly vulnerable to the effects of obesity [59]. Third, obesity-related cognitive deficits compromise the ability to learn and school performance, but also increase the risk of other unhealthy behaviours such as substance abuse, tobacco consumption, risky sex, and involvement in violence [60]. Last, but not least, many nonindustrialised countries have made an enormous effort to overcome undernutrition and preserve the full cognitive skills of their people. The transition from under- to overnutrition might be putting this achievement in jeopardy. Low-to-middle SES groups would be at higher risk of impaired expression of cognitive abilities due to greater exposure to overweight/obesity and risk factors.

### 4.3. Limitations and Strengths

These results support the connection between weight status and school performance. As most studies have been conducted in the developed world, one strength of the present study is that we provide evidence that is relevant for countries undergoing nutritional and epidemiological transitions. Second, we examined the relationship of adiposity markers and cognition using functional measures of cognition that were aimed at providing translational evidence that can be used for health promotion and brain protection in young populations. Furthermore, very few studies have examined the relation between weight status and school performance using adiposity markers other than BMI. Also, we used DXA to measure TFM. Yet, several limitations should be acknowledged when interpreting these results. Because our sample consisted of adolescents from low-to-middle SES, we are not able to extrapolate these results to the overall population of Chilean adolescents. Although a limitation, this SES bias makes our findings particularly relevant and thus could be taken as a strength for several reasons. The prevalence of overweightness/obesity is higher in adolescents of low-to-middle SES according to nationally conducted surveys [5,6]. Also, Chilean adolescents from these socioeconomic levels are more exposed to risk factors for excessive weight gain, such as unhealthy dietary habits and physical inactivity. Studies have shown that the negative effects of excess weight may disproportionately affect minorities and poor school children [61]. Last, the impact of SES on weight status and lifestyles could be critical during adolescence. Other limitations of this study should be mentioned and thus considered when interpreting our results. Although we controlled for sex, diet quality, PA status, family structure, and type of secondary education, we did not consider other important influences, such as school characteristics, potential learning disorders, or general motivational factors. Further studies should replicate this analysis in other populations of nonindustrialized countries. Finally, because the association between weight status and school performance does not imply causation, subsequent research should further investigate the effects of adiposity on cognitive health and educational outcomes. 

## 5. Conclusions

In Chilean adolescents of low-to-middle socioeconomic background, we found a negative and significant association of several fatness measures with school performance, independent of other influences, including diet quality and time allocation for PA. Having obesity, abdominal obesity, or high adiposity might be a potential risk factor for lower academic performance in males, but not always in females. When increased fatness is combined with unhealthy eating and physical inactivity, it becomes a risk factor for lower school performance in both males and females.

## Figures and Tables

**Table 1:** Descriptive statistics of male and female participants in the sample (*n* = 632).

	Total (*n* = 632)		Males (*n* = 330)		Females (*n* = 302)		*p* Value *
	Mean or *n*	(SD) or %	Mean or *n*	(SD) or %	Mean or *n*	(SD) or %	
**Chronological age**							
Age at assessment (year)	16.8	(0.3)	16.8	(0.3)	16.8	(0.2)	NS
Age at high school completion	18.5	(0.8)	18.5	(0.9)	18.4	(0.8)	NS
**Anthropometrics**							
Body-Mass Index (*z* score)	0.64	(1.2)	0.56	(1.2)	0.72	(1.1)	NS
Total Fat Mass (%)	29.0	(10.7)	22.3	(8.7)	36.2	(7.2)	<0.001
Waist circumference (cm)	81.9	(11.1)	80.9	(10.5)	81.1	(11.7)	NS
Obesity	86	13.6%	42	12.7%	44	14.6%	NS
Abdominal obesity	206	32.6%	61	18.5%	145	70.4%	<0.001 ^‡^
High adiposity	280	44.3%	115	34.7%	165	55.1%	<0.001 ^‡^
**School grades ^§^**							
9th (*n* = 632)	464.5	(99.6)	453.7	(95.6)	476.2	(100.1)	0.008
10th (*n* = 615)	468.5	(98.5)	457.7	(90.9)	480.1	(100.5)	0.011
11th (*n* = 576)	482.8	(95.9)	464.1	(90.1)	502.1	(102.2)	<0.001
12th (*n* = 571)	494.2	(98.4)	475.7	(89.9)	513.1	(103.1)	<0.001
Grade-Point Average (*n* = 571)	481.1	(92.3)	466.3	(82.7)	496.3	(98.8)	<0.001
**Socioeconomic background**							
Mother with incomplete HS	212	33.5%	114	32.5%	98	34.5%	NS ^‡^
Father with incomplete HS	176	27.9%	74	22.4%	102	33.8%	0.001 ^‡^
Fatherless family	250	39.6%	131	39.7%	119	39.4%	NS ^‡^
**Lifestyles**							
Unhealthy diet	132	20.9%	66	20.0%	66	21.9%	NS ^‡^
Physically inactive	367	58.1%	147	44.6%	220	72.9%	<0.001 ^‡^
**Type of secondary education**							
Academic	179	28.3%	85	25.8%	94	31.1%	NS ^‡^
Vocational	330	52.2%	176	53.3%	154	51.0%	
Adult	123	19.5%	69	20.9%	54	17.8%	
**Fe supplementation (6–12 months)**							
No added Fe	269	42.6%	148	44.5%	121	40.1%	NS ^‡^

* Two-tailed Student’s *t*-test was used for independent samples, except as indicated. ^‡^ Pearson’s chi-squared test. ^§^ School grades expressed as standardized score (scale 210–825), according to the Ministry of Education (Chile). Obesity: BMI*z* score >2 SD. Abdominal obesity: Waist Circumference ≥90 cm (males) or ≥80 cm (females). High adiposity: Total Fat Mass ≥25% (males) or ≥35% (females). Abbreviations: NS: not significant. HS: high school.

**Table 2:** Analysis of covariance (ANCOVA) measuring the effect of obesity and diet quality on school grades in male and female adolescents.

	**MALE PARTICIPANTS**										
	**Obesity (−)**					**Obesity (+)**					
	**Fair–to–Healthy Diet ^§^**		**Unhealthy Diet**			**Fair–to–Healthy Diet ^§^**			**Unhealthy Diet**		
	**Intercept**	**SE**	**Coefficient**	**SE**	***d***	**Coefficient**	**SE**	***d***	**Coefficient**	**SE**	***d***
			Model 1 ^a^								
9th (*n* = 330)	462.1 ***	6.2	−23.1 ^‡^	8.2	0.27	−39.6 *	19.6	0.49	−62.5 *	21.4	0.74
10th (*n* = 318)	464.8 ***	7.4	−21.9 ^‡^	6.6	0.25	−39.4 *	18.1	0.43	−75.6 *	28.2	0.70
11th (*n* = 292)	474.9 ***	6.6	−23.4 *	7.0	0.29	−47.8 *	16.3	0.59	−77.2 **	25.7	0.85
12th (*n* = 288)	485.0 ***	6.7	−25.9 **	4.2	0.32	−42.4 *	19.6	0.52	−56.2 **	15.9	0.77
HS–GPA (*n* = 288)	475.8 ***	5.5	−24.9 *	6.9	0.30	−42.7 *	17.3	0.62	−58.4 **	16.4	0.85
			Model 2 ^b^								
9th (*n* = 330)	466.9 ***	9.2	−22.9 ^‡^	9.2	0.25	−35.6 *	15.3	0.32	−67.5 *	23.9	0.58
10th (*n* = 318)	474.9 ***	9.7	−23.1 ^‡^	9.6	0.24	−37.7 *	13.1	0.34	−77.9 *	28.2	0.67
11th (*n* = 292)	475.6 ***	10.6	−25.9 *	8.9	0.32	−49.1 *	16.3	0.44	−78.6 **	26.7	0.66
12th (*n* = 288)	488.2 ***	9.7	−25.9 *	8.2	0.33	−42.4 *	17.6	0.40	−57.2 **	19.3	0.51
HS–GPA (*n* = 288)	480.5 ***	9.3	−25.2 *	8.7	0.33	−43.1 *	17.1	0.42	−61.4 **	19.1	0.53
	**FEMALE PARTICIPANTS**										
	**Obesity (−)**					**Obesity (+)**					
	**Fair–to–Healthy Diet ^§^**		**Unhealthy Diet**			**Fair–to–Healthy Diet ^§^**			**Unhealthy Diet**		
	**Intercept**	**SE**	**Coefficient**	**SE**	***d***	**Coefficient**	**SE**	***d***	**Coefficient**	**SE**	***d***
			Model 1 ^a^								
9th (*n* = 330)	485.9 ***	7.2	−19.8	12.2	0.15	−33.1	21.8	0.26	−50.3 *	22.4	0.57
10th (*n* = 318)	492.0 ***	9.1	−21.4	11.6	0.18	−35.2	20.5	0.26	−49.0 *	26.9	0.55
11th (*n* = 284)	513.5 ***	6.7	−26.6 *	8.8	0.33	−38.8 *	19.1	0.37	−50.4 **	19.1	0.60
12th (*n* = 281)	528.4 ***	8.1	−19.7 ^‡^	9.9	0.25	−41.4 *	19.6	0.36	−59.6 **	19.7	0.53
HS–GPA (*n* = 281)	507.6 ***	7.1	−24.1 *	10.9	0.30	−39.9 *	21.6	0.38	−51.5 *	18.4	0.43
			Model 2 ^b^								
9th (*n* = 330)	485.9 ***	10.2	−20.5	12.2	0.15	−33.1	22.8	0.23	−56.3 *	18.0	0.45
10th (*n* = 318)	492.0 ***	9.7	−24.9 ^‡^	12.1	0.17	−37.9	22.5	0.25	−41.0 *	16.9	0.33
11th (*n* = 284)	508.2 **	8.8	−29.6 *	9.5	0.30	−41.1 *	19.0	0.37	−55.9 *	17.1	0.40
12th (*n* = 281)	518.5 ***	10.3	−28.1	10.2	0.15	−44.4 *	19.7	0.37	−59.6 *	18.7	0.42
HS–GPA (*n* = 281)	507.6 ***	10.9	−25.7	10.8	0.19	−35.9	22.6	0.26	−52.0 *	15.4	0.38

Obesity (+): BMI*z* > 2 SD for age and sex. School grades expressed as standardized score (scale 210–825), according to the Ministry of Education (Chile). ^a^ Model 1 is unadjusted. ^b^ Model 2 is adjusted for SES (parental education, family structure), type of secondary education, and iron supplementation in infancy. ^§^ Participants with obesity (−) (BMI*z* score < 2 SD) and a fair-to-healthy diet are the reference group. Coefficients are the mean difference between a given category and the reference group. ^‡^ Trend towards significance (*p* < 0.10). * *p* < 0.05; ** *p* < 0.01; *** *p* < 0.001. Cohen’s *d* statistics were estimated accounting for different sample sizes. Abbreviation: HS–GPA: high school grade–point average.

**Table 3:** Analysis of covariance (ANCOVA) measuring the effect of abdominal obesity and diet quality on school grades in male and female adolescents.

	**MALE PARTICIPANTS**										
	**Abdominal Obesity (** **−)**					**Abdominal Obesity (+)**					
	**Fair–to–Healthy Diet ^§^**		**Unhealthy Diet**			**Fair–to–Healthy Diet ^§^**			**Unhealthy Diet**		
	**Intercept**	**SE**	**Coefficient**	**SE**	***d***	**Coefficient**	**SE**	***d***	**Coefficient**	**SE**	***d***
			Model 1 ^a^								
9th (*n* = 330)	465.4 ***	6.6	−25.6 ^‡^	15.2	0.27	−43.6 *	13.8	0.41	−87.7 **	19.4	0.73
10th (*n* = 318)	468.5 ***	6.4	−32.9 *	11.7	0.41	−49.9 *	12.7	0.40	−88.9 **	18.2	0.73
11th (*n* = 292)	476.2 ***	6.6	−26.4 *	12.9	0.34	−52.4 *	11.0	0.53	−87.2 **	18.7	0.74
12th (*n* = 288)	485.5 ***	7.7	−28.5 *	12.5	0.36	−50.8 *	11.6	0.50	−86.4 **	19.1	0.73
HS–GPA (*n* = 288)	478.0 ***	5.7	−29.5 *	13.5	0.37	−54.3 *	13.3	0.58	−82.9 **	19.4	0.69
			Model 2 ^b^								
9th (*n* = 330)	460.0 ***	9.6	−25.6	13.2	0.19	−44.1 *	18.8	0.39	−89.0 **	21.4	0.69
10th (*n* = 318)	478.0 ***	9.4	−33.5 *	12.7	0.37	−47.4 *	16.7	0.43	−88.9 **	22.2	0.69
11th (*n* = 292)	476.5 ***	9.6	−31.4 *	13.9	0.37	−50.4 *	15.0	0.41	−89.2 **	19.9	0.69
12th (*n* = 288)	488.1 ***	10.7	−30.5	16.5	0.21	−48.1 *	18.6	0.39	−87.7 **	19.1	0.67
HS–GPA (*n* = 288)	482.2 ***	9.3	−33.5 *	12.5	0.37	−53.9 *	13.9	0.45	−83.9 **	20.1	0.64
	**FEMALE PARTICIPANTS**										
	**Abdominal Obesity (** **−)**					**Abdominal Obesity (+)**					
	**Fair–to–Healthy Diet ^§^**		**Unhealthy Diet**			**Fair–to–Healthy** **Diet ^§^**			**Unhealthy Diet**		
	**Intercept**	**SE**	**Coefficient**	**SE**	***d***	**Coefficient**	**SE**	***d***	**Coefficient**	**SE**	***d***
			Model 1 ^a^								
9th (*n* = 330)	505.5 ***	9.7	−32.8 *	12.2	0.30	−47.1 **	15.8	0.36	−74.0 ***	19.6	0.55
10th (*n* = 318)	503.5 ***	10.3	−36.2 ^‡^	15.7	0.24	−49.6 *	17.9	0.33	−69.8 ***	18.8	0.59
11th (*n* = 284)	532.1 ***	10.1	−31.4 ^‡^	14.6	0.27	−56.2 *	18.5	0.40	−88.0 ***	18.9	0.75
12th (*n* = 281)	545.5 ***	9.7	−32.6 *	12.8	0.37	−48.8 *	18.1	0.34	−91.4 ***	19.1	0.76
HS–GPA (*n* = 281)	525.0 ***	9.8	−36.9 **	11.2	0.38	−58.6 **	13.2	0.42	−84.6 ***	19.2	0.68
			Model 2 ^b^								
9th (*n* = 330)	512.7 ***	12.7	−35.8 ^‡^	15.0	0.27	−49.1 *	18.8	0.33	−70.0 ***	21.6	0.51
10th (*n* = 318)	504.5 ***	12.5	−36.0	19.7	0.19	−51.4 *	20.9	0.33	−73.8 ***	21.9	0.52
11th (*n* = 284)	528.1 ***	12.1	−35.4 ^‡^	18.6	0.25	−58.0 *	20.5	0.39	−90.3 ***	21.9	0.69
12th (*n* = 281)	538.9 ***	11.7	−37.6 *	18.8	0.32	−50.3 *	19.6	0.34	−91.4 ***	20.1	0.69
HS–GPA (*n* = 281)	520.2 ***	11.8	−38.1 *	14.2	0.35	−60.6 **	17.2	0.40	−87.0 ***	21.2	0.64

Abdominal obesity (+): waist circumference ≥90 cm in males and ≥80 cm in females. School grades expressed as standardized score (scale 210–825), according to the Ministry of Education (Chile). ^a^ Model 1 is unadjusted. ^b^ Model 2 is adjusted for socioeconomic background (parental education, family structure), type of secondary education, age of high school completion, and iron supplementation in infancy. ^§^ Participants with abdominal obesity (−) and a fair-to-healthy diet are the reference group. Coefficients are the mean difference between a given category and the reference group. ^‡^ Trend towards significance (*p* < 0.10). * *p* < 0.05; ** *p* < 0.01; *** *p* < 0.001. Cohen’s *d* statistics were estimated accounting for different sample sizes. Abbreviations: HS–GPA: high school grade–point average.

**Table 4:** Analysis of covariance (ANCOVA) measuring the effect of high adiposity and diet quality on school grades in male and female adolescents.

	**MALE PARTICIPANTS**										
	**High Adiposity (** **−)**					**High Adiposity (+)**					
	**Fair–to–Healthy Diet ^§^**		**Unhealthy Diet**			**Fair–to–Healthy Diet ^§^**			**Unhealthy Diet**		
	**Intercept**	**SE**	**Coefficient**	**SE**	***d***	**Coefficient**	**SE**	***d***	**Coefficient**	**SE**	***d***
			Model 1 ^a^								
9th (*n* = 330)	462.4 ***	7.2	−29.9 *	9.2	0.35	−39.6 *	13.0	0.39	−65.7 **	22.4	0.61
10th (*n* = 318)	464.0 ***	6.4	−33.9 **	8.6	0.39	−46.7 *	12.7	0.41	−65.9 **	21.2	0.62
11th (*n* = 292)	467.8 ***	6.6	−34.4 **	9.9	0.39	−54.3 *	14.0	0.52	−77.2 **	23.7	0.64
12th (*n* = 288)	481.3 ***	7.7	−33.5 **	9.7	0.38	−52.3 **	11.6	0.51	−76.4 **	24.1	0.69
HS–GPA (*n* = 288)	473.0 ***	7.5	−33.9 *	10.9	0.37	−56.3 **	12.3	0.56	−72.9 **	20.4	0.68
			Model 2 ^b^								
9th (*n* = 330)	466.9 ***	11.2	−25.9 ^‡^	14.2	0.27	−40.1 *	16.0	0.36	−66.8 **	23.4	0.58
10th (*n* = 318)	474.7 ***	7.4	−29.0 *	13.0	0.33	−43.7 *	16.7	0.36	−67.1 **	23.2	0.56
11th (*n* = 292)	469.6 ***	8.6	−31.8 *	12.9	0.33	−50.0 *	17.9	0.39	−72.2 **	23.7	0.58
12th (*n* = 288)	481.3 ***	7.7	−29.9 *	12.7	0.33	−47.9 **	17.6	0.39	−74.4 **	24.1	0.62
HS–GPA (*n* = 288)	478.4 ***	8.5	−30.1 *	12.9	0.30	−49.3 **	17.3	0.39	−75.9 **	21.4	0.65
	**FEMALE PARTICIPANTS**										
	**High Adiposity (** **−)**					**High Adiposity (+)**					
	**Fair-to-Healthy Diet ^§^**		**Unhealthy Diet**			**Fair-to-Healthy ^§^**			**Unhealthy Diet**		
	**Intercept**	**SE**	**Coefficient**	**SE**	***d***	**Coefficient**	**SE**	***d***	**Coefficient**	**SE**	***d***
			Model 1 ^a^								
9th (*n* = 330)	501.9 ***	10.2	−39.2 *	13.2	0.40	−41.7 *	18.8	0.38	−61.3 *	19.4	0.45
10th (*n* = 318)	503.5 ***	11.1	−31.4 *	14.6	0.30	−40.6 *	16.5	0.39	−68.0 **	18.9	0.48
11th (*n* = 284)	530.4 ***	11.3	−32.0 *	13.8	0.32	−47.8 *	16.1	0.46	−72.4 **	18.1	0.49
12th (*n* = 281)	544.4 ***	11.4	−35.9 *	14.3	0.35	−52.4 *	18.6	0.50	−79.6 **	19.7	0.56
HS–GPA (*n* = 281)	524.3 ***	10.7	−37.0 **	13.2	0.39	−58.5 *	18.6	0.58	−64.6 **	19.4	0.52
			Model 2 ^b^								
9th (*n* = 330)	509.6 ***	11.2	−30.0	18.2	0.22	−31.7	24.9	0.22	−64.0 *	22.4	0.45
10th (*n* = 318)	503.5 ***	12.5	−29.4	17.6	0.23	−32.6	25.5	0.22	−72.1 **	21.9	0.48
11th (*n* = 284)	526.8 ***	12.3	−28.0	17.8	0.24	−36.8 ^‡^	25.1	0.27	−73.1 **	22.1	0.47
12th (*n* = 281)	537.9 ***	12.4	−36.9 *	16.9	0.30	−52.4*	23.6	0.41	−78.0 **	22.7	0.50
HS–GPA (*n* = 281)	521.3 ***	12.7	−35.0 *	16.2	0.30	−55.5*	22.6	0.45	−67.0 **	23.7	0.43

High adiposity (+): Total Fat Mass ≥25% in males and ≥35% in females. School grades expressed as standardized score (scale 210–825), according to the Ministry of Education (Chile). ^a^ Model 1 is unadjusted. ^b^ Model 2 is adjusted for SES (parental education, family structure), type of secondary education, and iron supplementation in infancy. ^§^ Participants with high adiposity (−) and a fair-to-healthy diet are the reference group. Coefficients are the mean difference between a given category and the reference group. ^‡^ Trend towards significance (*p* < 0.10). * p < 0.05; ** p < 0.01; *** p < 0.001. Cohen’s *d* statistics were estimated accounting for different sample sizes. Abbreviations: HS–GPA: high school grade–point average.

**Table 5:** Analysis of covariance (ANCOVA) measuring the effect of obesity and time allocation for exercise on school grades in male and female adolescents.

	**MALE PARTICIPANTS**										
	**Obesity (** **−)**					**Obesity (+)**					
	**Physically Active ^§^**		**Physically Inactive**			**Physically Active ^§^**			**Physically Inactive**		
	**Intercept**	**SE**	**Coefficient**	**SE**	***d***	**Coefficient**	**SE**	***d***	**Coefficient**	**SE**	***d***
			Model 1 ^a^								
9th (*n* = 330)	454.2 ***	7.2	−17.5 *	5.2	0.22	−26.4 *	10.6	0.41	−44.5 **	14.4	0.47
10th (*n* = 318)	469.0 ***	7.4	−20.6 *	5.6	0.22	−26.9 *	11.1	0.33	−36.0 *	13.2	0.40
11th (*n* = 292)	458.4 ***	7.5	−21.4 *	5.6	0.26	−20.8	11.1	0.27	−46.2 **	14.1	0.56
12th (*n* = 288)	470.4 ***	6.7	−21.1 *	5.2	0.29	−28.4 *	11.6	0.41	−42.6 *	14.7	0.41
HS-GPA (*n* = 288)	463.6 ***	6.5	−21.9 *	4.9	0.30	−21.9	10.6	0.30	−47.2 **	15.4	0.50
			Model 2 ^b^								
9th (*n* = 330)	459.9 ***	8.6	−18.2	6.6	0.18	−28.1 *	13.1	0.30	−47.9 **	16.6	0.50
10th (*n* = 318)	466.0 ***	9.0	−22.1 *	7.4	0.20	−27.3 *	12.5	0.30	−44.7 **	16.1	0.47
11th (*n* = 292)	459.6 ***	8.5	−20.4 *	7.7	0.20	−24.1 *	11.8	0.28	−46.1 **	16.7	0.49
12th (*n* = 288)	468.4 ***	6.7	−21.9 *	6.3	0.28	−27.4 *	12.8	0.34	−45.6 *	17.8	0.51
HS-GPA (*n* = 288)	467.5 ***	7.8	−19.5 *	6.2	0.24	−28.4 *	12.6	0.30	−44.5 *	16.4	0.47
	**FEMALE PARTICIPANTS**										
	**Obesity (** **−)**					**Obesity (+)**					
	**Physically Active ^§^**		**Physically Inactive**			**Physically Active ^§^**			**Physically Inactive**		
	**Intercept**	**SE**	**Coefficient**	**SE**	***d***	**Coefficient**	**SE**	***d***	**Coefficient**	**SE**	***d***
			Model 1 ^a^								
9th (*n* = 330)	473.4 ***	7.7	−18.1 *	5.3	0.22	−21.5	12.8	0.19	−48.7 **	14.4	0.58
10th (*n* = 318)	478.7 ***	7.6	−16.4 *	5.6	0.21	−22.7	12.0	0.19	−43.0 **	16.9	0.53
11th (*n* = 284)	458.4 ***	7.5	−17.3 *	5.6	0.21	−22.8 *	10.1	0.24	−40.4 **	13.1	0.43
12th (*n* = 281)	514.0 ***	6.7	−18.2 *	4.2	0.24	−23.8 *	10.6	0.28	−45.6 **	13.7	0.50
HS-GPA (*n* = 281)	494.9 ***	6.5	−20.5 *	4.9	0.24	−28.4 *	11.6	0.32	−47.6 **	14.4	0.57
			Model 2 ^b^								
9th (*n* = 330)	480.0 ***	9.2	−17.1	5.3	0.17	−17.7	13.8	0.17	−45.7 *	17.4	0.41
10th (*n* = 318)	485.3 ***	9.4	−19.4 *	5.6	0.21	−20.1	13.5	0.18	−41.5 *	16.9	0.33
11th (*n* = 284)	504.8 ***	9.5	−18.3 *	4.7	0.21	−20.8	12.0	0.18	−44.4 **	16.4	0.36
12th (*n* = 281)	520.4 ***	9.7	−20.7 *	5.3	0.20	−29.4 *	12.5	0.27	−44.9 *	18.2	0.35
HS-GPA (*n* = 281)	501.5 ***	8.5	−19.1 *	4.9	0.20	−30.4 *	13.6	0.29	−44.6 *	17.6	0.40

Obesity (+): BMI*z* > 2 SD for age and sex. School grades expressed as standardized score (scale 210–825), according to the Ministry of Education (Chile). ^a^ Model 1 is unadjusted. ^b^ Model 2 is adjusted for SES (parental education, family structure), type of secondary education, and iron supplementation in infancy. ^§^ Participants with obesity (−) (BMI*z* score < 2 SD) and a fair-to-healthy diet are the reference group. Coefficients are the mean difference between a given category and the reference group. ^‡^ Trend towards significance (*p* < 0.10). * *p* < 0.05; ** *p* < 0.01; *** *p* < 0.001. Cohen’s *d* statistics were estimated accounting for different sample sizes. Abbreviations: HS–GPA: high school grade–point average.

**Table 6:** Analysis of covariance (ANCOVA) measuring the effect of abdominal obesity and time allocation for exercise on school grades in male and female adolescents.

	**MALE PARTICIPANTS**										
	**Abdominal Obesity (** **−)**					**Abdominal Obesity (+)**					
	**Physically Active ^§^**		**Physically Inactive**			**Physically Active ^§^**			**Physically Inactive**		
	**Intercept**	**SE**	**Coefficient**	**SE**	***d***	**Coefficient**	**SE**	***d***	**Coefficient**	**SE**	***d***
	Model 1 ^a^										
9th (*n* = 330)	460.9 ***	7.2	−18.7 *	4.2	0.22	−33.4 *	12.6	0.42	−59.2 *	11.4	0.74
10th (*n* = 318)	459.0 ***	7.4	−19.2 *	5.6	0.24	−33.5 *	12.1	0.42	−61.2 *	13.2	0.73
11th (*n* = 292)	462.8 ***	7.5	−16.3 *	5.6	0.20	−35.4 *	11.1	0.47	−60.7 *	12.8	0.73
12th (*n* = 288)	474.4 ***	6.7	−16.4 *	4.2	0.20	−30.4 *	7.6	0.41	−59.8 *	12.7	0.82
HS-GPA (*n* = 288)	468.7 ***	6.5	−18.9 *	5.9	0.24	−35.6 *	11.6	0.47	−57.6 *	12.4	0.82
	Model 2 ^b^										
9th (*n* = 330)	465.7 ***	9.0	−15.9*	4.3	0.20	−35.4 *	13.6	0.36	−61.7 *	16.4	0.73
10th (*n* = 318)	470.0 ***	8.8	−17.8*	5.6	0.21	−33.5 *	13.1	0.36	−63.1 *	16.0	0.78
11th (*n* = 292)	462.6 ***	8.5	−15.3 ^‡^	5.9	0.19	−35.1 *	12.6	0.40	−60.2 *	18.0	0.77
12th (*n* = 288)	470.8 ***	7.7	−16.5 ^‡^	4.2	0.19	−28.9 *	13.6	0.31	−57.1 *	17.7	0.65
HS-GPA (*n* = 288)	471.6 ***	8.5	−19.5*	6.0	0.22	−37.0 *	12.6	0.40	−57.4 *	17.6	0.65
	**FEMALE PARTICIPANTS**										
	**Abdominal Obesity (** **−)**					**Abdominal Obesity (+)**					
	**Physically Active ^§^**		**Physically Inactive**			**Physically Active ^§^**			**Physically Inactive**		
	**Intercept**	**SE**	**Coefficient**	**SE**	***d***	**Coefficient**	**SE**	***d***	**Coefficient**	**SE**	***d***
	Model 1 ^a^										
9th (*n* = 302)	482.7 ***	9.1	−15.1*	6.2	0.22	−30.5 *	9.1	0.34	−55.5 *	12.7	0.67
10th (*n* = 297)	484.3 ***	7.4	−15.4*	6.6	0.23	−31.9 *	10.9	0.35	−57.5 *	12.9	0.69
11th (*n* = 284)	511.3 ***	8.5	−11.1	7.3	0.19	−32.8 *	11.1	0.38	−54.0 *	12.1	0.60
12th (*n* = 281)	518.6 ***	7.7	−13.9^‡^	7.2	0.19	−40.3 *	11.6	0.41	−56.6 *	12.7	0.69
HS-GPA (*n* = 281)	502.3 ***	6.5	−15.1*	6.1	0.32	−30.6 *	12.6	0.38	−48.8 *	11.4	0.69
	Model 2 ^b^										
9th (*n* = 302)	487.6 ***	10.7	−14.1	8.2	0.18	−31.5 *	13.5	0.32	−50.5 *	17.0	0.45
10th (*n* = 297)	490.3 ***	10.0	−15.4 *	6.6	0.23	−32.9 *	14.5	0.33	−54.5 *	17.9	0.53
11th (*n* = 284)	515.6 ***	10.4	−11.7	7.3	0.17	−34.3 *	14.3	0.35	−52.0 *	18.2	0.49
12th (*n* = 281)	524.5 ***	11.7	−13.0	7.2	0.17	−38.1 *	13.6	0.37	−54.1 *	18.3	0.54
HS-GPA (*n* = 281)	508.2 ***	9.9	−20.3 *	7.0	0.29	−36.5 *	14.6	0.35	−56.9 *	17.5	0.54

Abdominal obesity (+): waist circumference ≥90 cm in males and ≥80 cm in females. School grades expressed as standardized score (scale 210–825), according to the Ministry of Education (Chile). ^a^ Model 1 is unadjusted. ^b^ Model 2 is adjusted for socioeconomic background (parental education, family structure), type of secondary education, age of high school completion, and iron supplementation in infancy. ^§^ Participants with abdominal obesity (−) and a fair-to-healthy diet are the reference group. Coefficients are the mean difference between a given category and the reference group. ^‡^ Trend towards significance (*p* < 0.10). * *p* < 0.05; ** *p* < 0.01; *** *p* < 0.001. Cohen’s *d* statistics were estimated accounting for different sample sizes. Abbreviations: HS–GPA: high school grade–point average.

**Table 7:** Analysis of covariance (ANCOVA) measuring the effect of high adiposity and time allocation for exercise on school grades in male and female adolescents.

	**MALE PARTICIPANTS**										
	**High Adiposity (** **−)**					**High Adiposity (+)**					
	**Physically Active ^§^**		**Physically Inactive**			**Physically Active ^§^**			**Physically Inactive**		
	**Intercept**	**SE**	**Coefficient**	**SE**	***d***	**Coefficient**	**SE**	***d***	**Coefficient**	**SE**	***d***
			Model 1 ^a^								
9th (*n* = 330)	459.8 ***	7.2	−20.0	7.2	0.19	−37.0 *	8.6	0.42	−52.2 **	9.4	0.62
10th (*n* = 318)	457.9 ***	7.4	−28.1 ^‡^	6.6	0.26	−36.5 *	6.1	0.42	−58.2 **	7.8	0.77
11th (*n* = 292)	456.8 ***	7.5	−28.7 ^‡^	6.6	0.27	−40.4 *	9.1	0.47	−59.2 **	6.8	0.78
12th (*n* = 288)	473.3 ***	6.7	−28.4 *	6.2	0.30	−44.4 *	7.6	0.49	−53.8 **	9.3	0.73
HS-GPA (*n*=288)	466.7 ***	6.5	−27.9 *	6.9	0.31	−42.3 *	6.6	0.49	−58.7 **	7.1	0.79
			Model 2 ^b^								
9th (*n* = 330)	464.8 ***	9.7	−12.8	7.9	0.15	−43.7 *	9.0	0.33	−50.9 **	11.4	0.49
10th (*n* = 318)	469.4 ***	10.4	−17.5 ^‡^	6.6	0.20	−41.8 *	10.1	0.33	−55.7 **	10.8	0.55
11th (*n* = 292)	457.0 ***	10.5	−17.1 ^‡^	6.5	0.20	−46.0 *	9.7	0.40	−61.2 **	12.1	0.57
12th (*n* = 288)	469.7 ***	10.7	−19.4 *	6.2	0.29	−46.7 *	10.2	0.42	−59.8 **	11.0	0.57
HS-GPA (*n* = 288)	469.3 ***	9.8	−17.9 *	6.9	0.30	−43.0 *	11.9	0.42	−58.7 **	10.9	0.57
	**FEMALE PARTICIPANTS**										
	**High Adiposity (** **−)**					**High Adiposity (+)**					
	**Physically Active ^§^**		**Physically Inactive**			**Physically Active ^§^**			**Physically Inactive**		
	**Intercept**	**SE**	**Coefficient**	**SE**	***d***	**Coefficient**	**SE**	***d***	**Coefficient**	**SE**	***d***
			Model 1 ^a^								
9th (*n* = 302)	482.7 ***	9.1	−14.1 ^‡^	7.2	0.23	−19.5	9.9	0.25	−55.5 **	10.7	0.55
10th (*n* = 297)	484.3 ***	7.4	−14.4 ^‡^	7.2	0.24	−20.9	10.3	0.25	−52.5 **	10.9	0.57
11th (*n* = 284)	511.3 ***	8.5	−19.1 *	6.4	0.30	−27.8 *	10.1	0.39	−54.0 **	10.1	0.55
12th (*n* = 281)	518.6 ***	7.7	−17.9 *	7.2	0.32	−26.3 *	9.6	0.41	−58.6 **	11.7	0.64
HS-GPA (*n* = 281)	502.3 ***	6.5	−15.1 *	6.1	0.30	−25.6 *	10.6	0.40	−57.8 **	10.4	0.64
			Model 2 ^b^								
9th (*n* = 302)	491.3 ***	12.1	−16.1 ^‡^	7.0	0.23	−25.0	12.9	0.24	−53.1 **	13.3	0.49
10th (*n* = 297)	490.3 ***	13.1	−17.0 ^‡^	7.2	0.24	−26.9	13.9	0.23	−56.5 **	14.7	0.50
11th (*n* = 284)	519.3 ***	12.5	−20.5 *	7.4	0.30	−27.1	13.1	0.25	−61.0 **	15.9	0.52
12th (*n* = 281)	469.7 ***	10.7	−23.0 *	8.2	0.31	−34.3 *	11.2	0.34	−59.8 **	15.8	0.52
HS-GPA (*n* = 281)	514.1 ***	9.5	−15.1 ^‡^	7.5	0.23	−32.9 *	9.6	0.35	−64.7 **	14.1	0.57

High adiposity (+): Total Fat Mass ≥25% in males and ≥35% in females. School grades expressed as standardized score (scale 210–825), according to the Ministry of Education (Chile). ^a^ Model 1 is unadjusted. ^b^ Model 2 is adjusted for SES (parental education, family structure), type of secondary education, and iron supplementation in infancy. ^§^ Participants with high adiposity (−) and a fair-to-healthy diet are the reference group. Coefficients are the mean difference between a given category and the reference group. ^‡^ Trend towards significance (*p* < 0.10). * p < 0.05; ** p < 0.01; *** p < 0.001. Cohen’s *d* statistics were estimated accounting for different sample sizes. Abbreviations: HS–GPA: high school grade–point average.

## Supplementary Materials

The following are available online at http://www.mdpi.com/2072-6643/10/9/1133/s1: Table S1: Comparison of participants included and excluded in the current analysis.
