# Supplementary material for: Increased Adiposity as a Potential Risk Factor for Lower Academic Performance: A Cross-Sectional Study in Chilean Adolescents from Low-to-Middle Socioeconomic Background
Source: Nutrients. 2018 Aug 21;10(9):1133. doi: 10.3390/nu10091133 (PMC6165189; doi:10.3390/nu10091133)
Supplement: Supplementary file 1 [file nutrients-10-01133-s001.pdf]

SUPPLEMENTARY MATERIAL

Table S1 Comparison of participants included and excluded in the current analysis

	Total sample (*n*=679)		Enrolled in high school (*n*=632)		Non-enrolled in high school (*n*=47)		*P* value*
	Mean or *n*	(SD) or %	Mean or *n*	(SD) or %	Mean or *n*	(SD) or %	
**Chronological age**							
Age at assessment (y)	16.8	(0.2)	16.8	(0.1)	16.9	(0.3)	NS
**Sex**							
Male	357	52.6%	330	52.2%	27	57.5%	NSǂ
**Anthropometrics**							
Body-Mass Index (*z* score)	0.65	(1.2)	0.64	(1.2)	0.81	(1.5)	NS
Waist circumference (cm)	81.3	(11.3)	81.0	(11.1)	84.9	(2.1)	0.024
Total Fat Mass (%)	29.0	(10.7)	29.0	(10.6)	29.4	(10.9)	NS
Weight status							
Excess weight	262	38.6%	241	38.1%	21	44.7%	NSǂ
Abdominal obesity	226	33.3%	206	32.6%	20	42.6%	NSǂ
High adiposity	301	44.3%	280	44.3%	21	43.5%	NSǂ
**Parental education**							
Mother's: incomplete secondary	240	35.4%	212	33.5%	28	60.9%	0.044ǂ
Father's: incomplete secondary	192	28.3%	176	27.9%	26	34.8%	NSǂ
**Lifestyles**							
Unhealthy eating	151	22.3%	132	20.9%	19	40.4%	0.002ǂ
Physically inactive	403	59.4%	367	58.1%	36	76.6%	0.013ǂ
**Family structure**							
Fatherless home	274	40.4%	250	39.6%	24	51.1%	NSǂ
**Iron supplementation (6-12 mo)**							
No-added Fe	286	42.2%	269	42.6%	17	37.0%	NSǂ

* Two-tailed Student's *t* test for independent samples except as indicated. ǂ Pearson's Chi^2. § School grades expressed as standardized score, according to the Ministry of Education (Chile). Excess weight: BMI*z* >2 SD. Abdominal obesity: WC >90 cm and >80 cm in males and females, respectively. High adiposity: Total Fat Mass ≥25% and ≥35%, respectively.
